# The Art of Seeing

**Published:** 1901-06-08

**Authors:** 


### The Hospital

A Journal of

The Medical Sciences and Hospital Administration.

Vol. XXX.—No. 767.] Edited by Sir Henry Burdett, K.C.B., and Solomon C. Smith, M.D., M.R.C.P. [June 8, 1901.

### The Art of Seeing.

The eye is the organ of vision. At least, so we are brought up to believe, and we have no doubt that the general public does thoroughly believe that what they see with is their eyes and nothing else. Mr. Brudenell Carter then did well, in speaking before the Society of Arts the other day, to show in the first place how widely extended is the mechanism, and how complex are the acts which are involved in that sort of perfect vision which is wanted by the soldier, and next, how largely sight can be improved by practice. Starting with the general statement that as an optical instrument the eye may be regarded as a camera capable of throwing an accurate image of the object upon the retina, he went on to show that the retina on which the image is received is a mosaic of hexagons, each of which represents a nerve ending, and that the acuity of vision, so far as form vision is concerned, depends ultimately upon the size of these hexagons. The more minute the bit of the picture which falls upon a separate nerve ending, and the more accurately we are able to distinguish between the stimulation of each separate nerve, the more minutely accurate is our vision likely to be. Thus there are two factors in the nerve element of perfect vision; one the size of the hexagonal endings of the cones and rods—a matter which we cannot alter—and the other the power of distinguishing between separate impressions—a power which, like tactile sensibility, is capable of almost indefinite improvement by practice.

From lack of opportunity of looking at small and distant objects—that is, objects subtending the very smallest division of the retina which is capable of separate stimulation—town dwellers lose the faculty of fine definition. If the eye is to be made to see the smallest object that, from its construction, it is capable of seeing, it must be practised on objects under small visual angles, and must so be taught to disentangle the images formed at the very limit of visibility.

But to get the best out of our eyes the ready and active co-operation of other nervous structures besides those within the eyeball is required. Quickness of perception, as well as acuteness of vision, is of great importance, and especially to the soldier. Mr. Carter illustrated what he meant by referring to the manner in which a child or an illiterate person reads a printed page. Such a person receives a perfect retinal image, and in a sense he sees the words perfectly, but he is not able to recognise them at a glance; he has to dwell upon each of them in succession, and, so to say, to see it slowly, with the result that he cannot look forward along the line and perceive what is coming with sufficient rapidity to modulate his voice in accordance with the meaning of the words. His eyes see each word just as well as a skilful reader would, but his consciousness responds slowly to the visual impression. There is a mental as well as a retinal end to the nervous chain engaged in vision. As he improves in reading by practice it is not his sight that improves but his power of responding quickly to visual images of a kind which he has been accustomed to observe and has learned to recognise. There are many games which give good training in quickness of sight, one of the best being cricket. Batting to a fast bowler is, according to Mr. Carter, an admirable exercise for the purpose.

Considerable interest attaches to what Mr. Brudenell Carter said as to the important aid to keen and rapid sight which is given by the possession of an acute and cultivated sense of colour. In order to estimate the value of the colour sense we must remember that mimicry of the general colour effect of natural objects forms the chief defence of many wild creatures against their enemies. Nothing but keenness of colour sense renders it possible to see a trout when looking directly down upon its back against the bottom of a pebbled stream, or to see a distant deer against the greys and greens and browns of Highland scenery, and this sense, like others, is capable of almost unlimited improvement by practice and cultivation. The man who can only see by outlines is constantly at a disadvantage as compared with one who is sensitive to the slightest difference in tint. It is satisfactory to find that Mr. Carter does not consider that non-use, even up to middle age, involves any permanent incapacity. "I feel confident," he says, "that ninety-nine English soldiers out of a hundred, after a fortnight's exercise on the veldt, would see anything that the Boers themselves could see and would interpret appearances as quickly." But to regain this power they must practise.

Another matter which is of great importance in attaining useful vision, especially for military purposes, is the cultivation of lateral vision. Acuteness of lateral depends largely upon sensibility of the lateral portions of the retina, but it also depends upon the quickness and exactness with which the eyes can move over the field, towards any doubtful object whose image may fall on the lateral portion of the retina, and spring back again with certainty and exactness to the spot on which they were fixed before. It is an everyday experience to find people with their eyes so fixed upon certain objects that they are absolutely blind to all else. "A man trained in woodcraft, on the other hand, would never allow his eyes to rest continuously upon anything . . . and would constantly range over every portion of the field that he desired to watch."

For perfect vision then we require a combination of various attributes, accurate and rapid perception of form, quick recognition of slight differences of colour, rapid fixation, comparatively acute sensibility of the lateral portions of the retina, quickness and exactitude in the action of the external muscles of the eye, and such activity of mental processes as shall at once associate the actual impression on the retina with things seen before. The optical portion of the visual apparatus is important, and indeed is essential; but it is not everything.

